# Effect of transannular interaction on the redox-potentials in a series of bicyclic quinones

**DOI:** 10.1186/1860-5397-2-26

**Published:** 2006-12-08

**Authors:** Grigoriy Sereda, Jesse Van Heukelom, Miles Koppang, Sudha Ramreddy, Nicole Collins

**Affiliations:** 1 The University of South Dakota, Department of Chemistry 414 E. Clark St., Vermillion, SD 57069, USA

## Abstract

**Background:**

Better understanding of the transannular influence of a substituent on the redox-potentials of bicyclo[2.2.2]octane-derived quinones will help in the design of new compounds with controlled biological activity. However, attempts to directly relate the reduction potentials of substituted triptycene-quinones to the electronic effects of substituents are often unsuccessful.

**Results:**

First and second redox-potentials of a series of bicyclic quinones are compared to computed energies of their LUMO, LUMO+1, and energies of reduction. Transannular influence of substituent on the redox-potentials is rationalized in terms of MO theory. Acetoxy-substituents in the 5,8-positions of the triptycene-quinone system selectively destabilize the product of the two-electron reduction.

**Conclusion:**

We have shown that first redox-potentials of substituted bicyclic quinones correlate with their calculated LUMO energies and the energies of reduction. The second redox-potentials correlate with calculated LUMO+1 energies. As opposed to the LUMO orbitals, the LUMO+1 orbital coefficients are weighted significantly on the non-quinone part of the bicyclic system. This accounts for: (1) significantly larger substituent effect on the second redox-potentials, than on the first redox-potentials; (2) lack of stability of the product of two electron reduction of 5,8-diacetoxy-9,10-dihydro-9,10-[1,2]benzenoanthracene-1,4-dione **5**.

## Background

**Figure 1:**
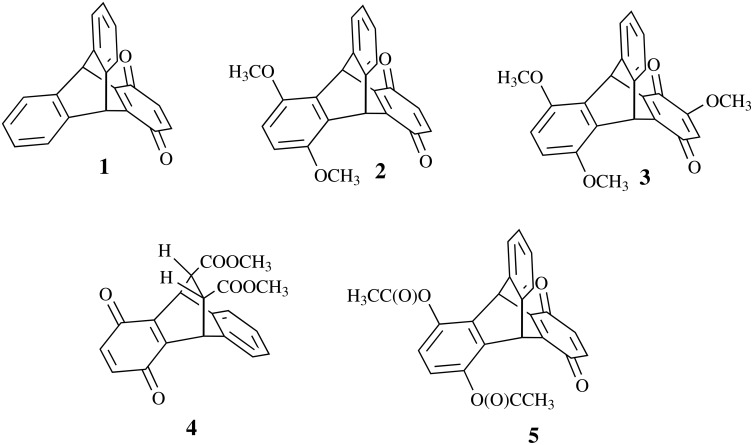
Bicyclic quinones explored for the transannular interaction.

It has been shown that 9,10-dihydro-9,10-[1,2]benzenoanthracene-1,4-dione (triptycene-quinone, **1**) exhibits anti-leukemia activity, comparable with activity of substituted triptodiquinones [1]. One of the reasons for such activity is believed to be caused by the oxidizing properties of the quinone ring [1]. A recent study has revealed significant anti-inflammatory activity of the substituted triptycene-quinones **2** and **3** (Figure 1), which is also believed to be linked to the free radical redox-processes, involving triptycene-quinones and reactive oxygen species [2]. Better understanding of the transannular influence of a substituent on the redox-potentials of bicyclo[2.2.2]octane-derived quinones will help in the design of new compounds with controlled biological activity. However, attempts to directly relate the reduction potentials of substituted triptycene-quinones to the electronic effects of substituents are often unsuccessful. Thus, the negative shift of the reduction potential, caused by two methoxy-groups at the 5,8-positions (compound **2**), was surprisingly only half the decrease caused by the 6,7-methoxy-groups, which are more distant from the quinone fragment [3].

Here we report cyclic voltammetric data and DFT (Density Functional Theory) calculations of five bicyclic quinones **1–5** (Figure 1) with the purpose to relate their redox-potentials to the calculated parameters and to the nature and positions of substituents in the bicyclic system.

## Results and Discussion

Accurate computational prediction of redox-potentials requires comparison of energies for both the starting quinone and its reduced forms. The open-shell nature of the reduced species and often the necessity to take into account solvation makes the prediction of the redox-potentials a challenging and time consuming computational problem. However, Koopmans' theorem [4] enabled us to relate redox-potentials of bicyclic quinones with their LUMO energies, which characterize solely the starting compound. Despite the neglected orbital relaxation that immediately follows the reduction, such correlations have proved to be an efficient tool for prediction of redox-potentials of anthracyclines [5], substituted anthracenes [6], and oligothiophenes [7].

**Figure 2:**
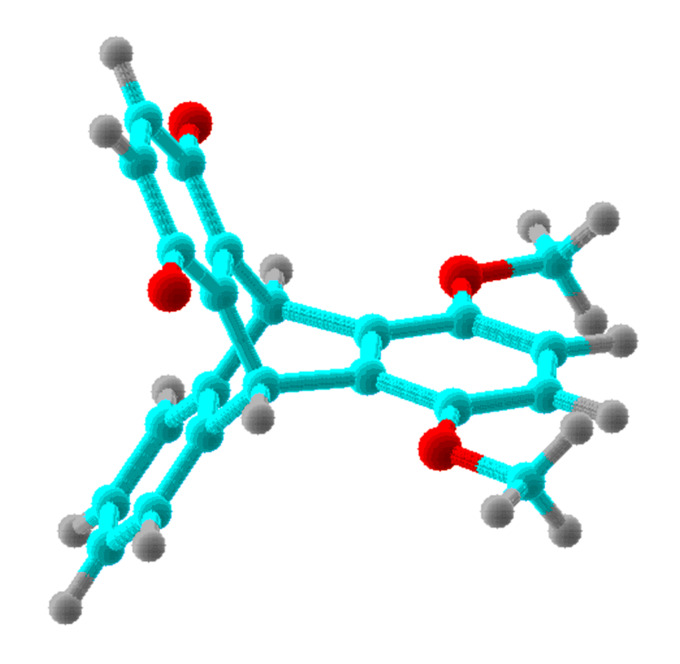
Conformation **A** of compound **2**.

**Figure 3:**
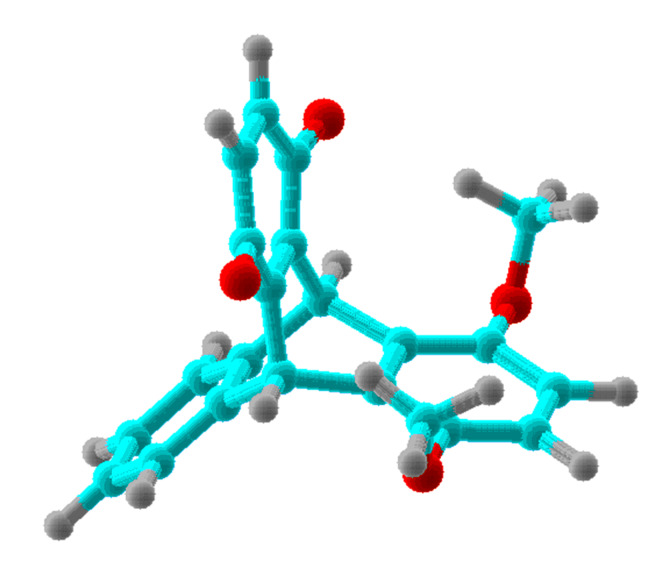
Conformation **B** of compound **2**.

For all chemical species, the computations were performed for the global minimum conformation. These conformations for the methoxy-derivatives **2, 3, 2^·−^**, and **3^·−^** correspond to the α-methyl groups, symmetric with respect to the plane of the benzene ring (conformation **A**, Figure 2). In addition, we present computational results for the alternative conformation of **2** with two methyl groups oriented toward the quinone ring (conformation **B**, Figure 3). To minimize steric repulsion in the alternative conformation of the trimethoxy-derivative **3**, only the methyl group remote from the quinone methoxy-group was oriented toward the quinone ring. These alternative conformations are marked with asterisk in the Table 1 and in the following text and Figures.

**Table 1:** Cyclic Voltammetry data for compounds **1**–**5**

**Compound**	**E_pr1_, V**	**E_pox1_, V**	**E^o'^_1_, V**	**E_pr2_, V**	**E_pox2_, V**	**E^o'^_2_, V**

**1**	-0.453	-0.369	-0.411	-1.065	-0.976	-1.0205
**2**	-0.435	-0.360	-0.398	-0.980	-0.894	-0.937
**3**	-0.550	-0.470	-0.510	-1.090	-0.994	-1.042
**4**	-0.441	-0.360	-0.401	-1.010	-0.923	-0.9665
**5**	-0.376	-0.296	-0.336	-0.804	-0.610	-0.707

**Figure 4:**
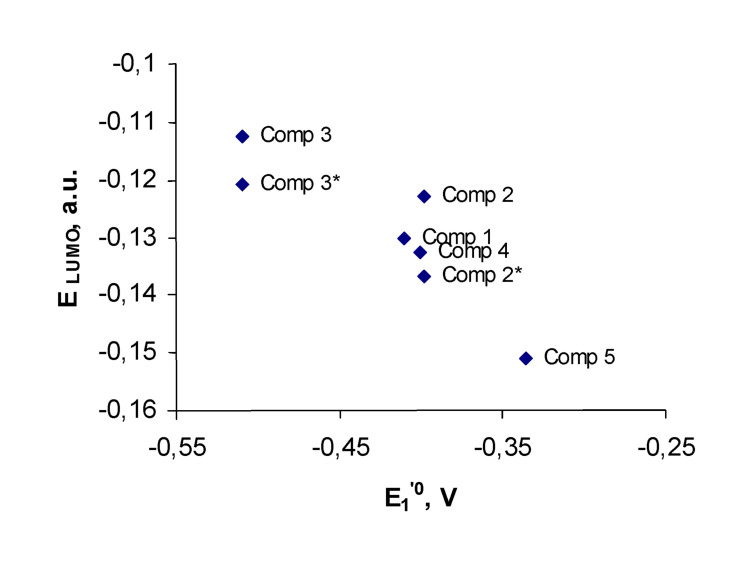
First formal redox-potentials of compounds **1**–**5** vs. their calculated LUMO energies.

The Figure 4 shows correlation between the first redox-potentials and calculated LUMO energies for compounds **1–5**.

The experimental redox-potential of the quinone **2** is 50 mV higher, than expected, based on this correlation and the calculated LUMO energy for its conformation **A**. This unexpectedly high redox-potential of **2** is consistent with the LUMO energy, calculated for the conformation **B** with two methyl groups, turned toward the quinone ring. This conformation is stabilized by weak H-bonds with the quinone carbonyl oxygens (2.5 A). Due to the lack of conjugation between the lone electron pairs of the methoxy groups and the benzene ring, conformation **B** is 5.5 kcal/mol higher in energy than conformation **A**. This value calculated for vacuum, can be greatly affected by solvation. Therefore, the energy difference may fall below the threshold that would warrant sufficient concentrations of the conformation **B** to account for the experimental redox-potential of **2**.

Yamamura and co-authors [3] also noticed that the reduction potential of the quinone **2** was higher than expected from the electronic effects of the methoxy-substituents. They explained this difference by the parallel alignment of the C-O-bond with the π-system of the benzene ring, which amplifies the inductive effect of the methoxy-group. In other words, quinone **2** assumes the conformation **B**. As we move from the conformation **A** to conformation **B**, changing of the C-C-O-C dihedral angle from 0° to 90° enhances the inductive effect of the methoxy-group and weakens its counterbalancing resonance effect. Therefore, our computations provide additional support for the assumption of Yamamura.

**Figure 5:**
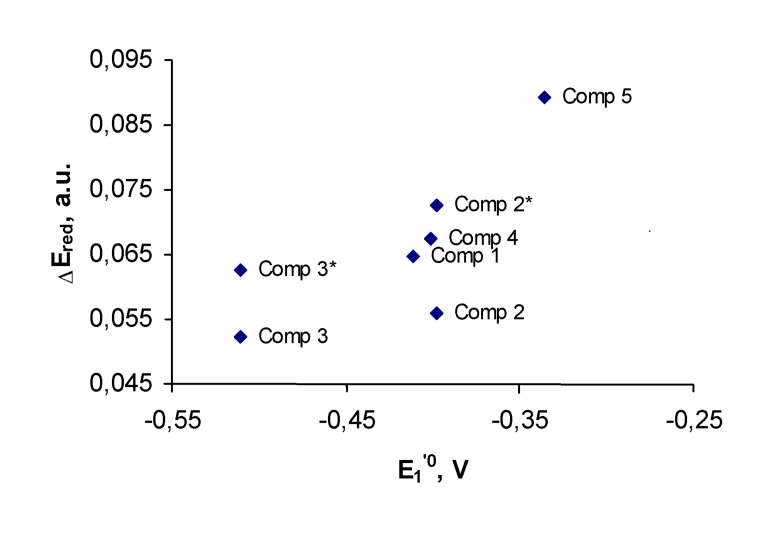
First formal redox-potentials of compounds **1**–**5** vs. their calculated energies of reduction.

For quinone **3**, both the global minimum and the alternative conformations fit well into the correlation (Figure 4). A more precise approach to prediction of redox-potential should involve comparison of energies of both the original quinone and its reduced form. Figure 5 shows correlation between the first redox-potentials and calculated energies of reduction for compounds **1–5**. The energies of reduction were calculated as a difference between the energy of the reduced form and the original quinone.

Due to the computational challenges of optimization open-shell structures **1^·−^–5^·−^**, correlation between first redox-potentials of quinones and their reduction energies takes significantly more computational time, but does not substantially improve the quality of prediction.

**Figure 6:**
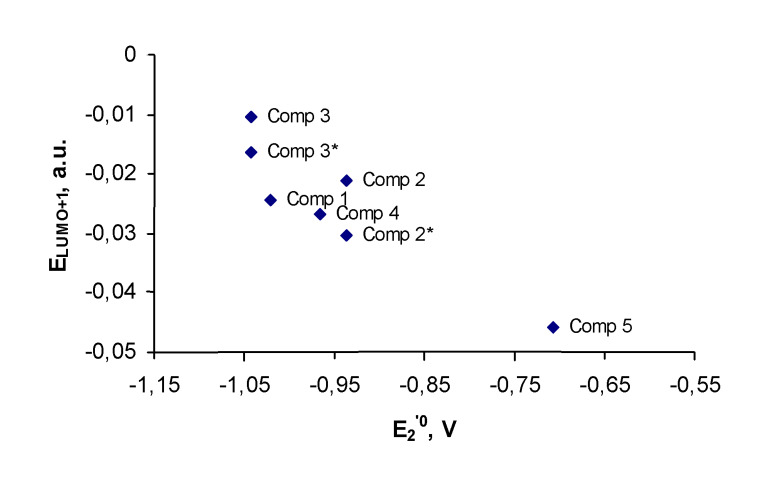
Second formal redox-potentials of compounds **1**–**5** vs. their calculated LUMO+1 energies.

As opposed to the first redox-potentials, we did not find any correlation between the second redox-potentials and the LUMO energies for the reduced species **1^·−^–5^·−^**, computed at the time permissible level of theory. This computational challenge may be partly due to the degenerate nature of the LUMO and LUMO+1 orbitals of **1^·−^–5^·−^**. However, the second redox-potentials can be easily predicted due to their correlation with the calculated LUMO+1 energies of the starting quinones, shown in Figure 6.

This correlation illustrates that similarly to the first redox-potentials, prediction of second redox-potentials should be performed with consideration of the conformation with the highest oxidation potential, which is conformation **B** for quinones **2** and **3**. The correlation on Figure 5 and Figure 6 demonstrate that the Koopmans' theorem provides us with the useful tool to evaluate both the first and the second redox-potentials for the series of bicyclic quinones.

It is worthwhile to note that substituents in the non-quinone ring exert significantly stronger influence (by the factor of 4 to 6, see Table 1 in the Experimental section) on the second redox-potential, than on the first potential. Contrary, the methoxy-group, attached to the quinone ring in the compound **3** has similar effect (about 0.1 V) on both the first and second redox-potentials. To understand the reason of such behavior, we need to consider the transannular orbital interaction in the quinones **1–5**.

**Figure 7:**
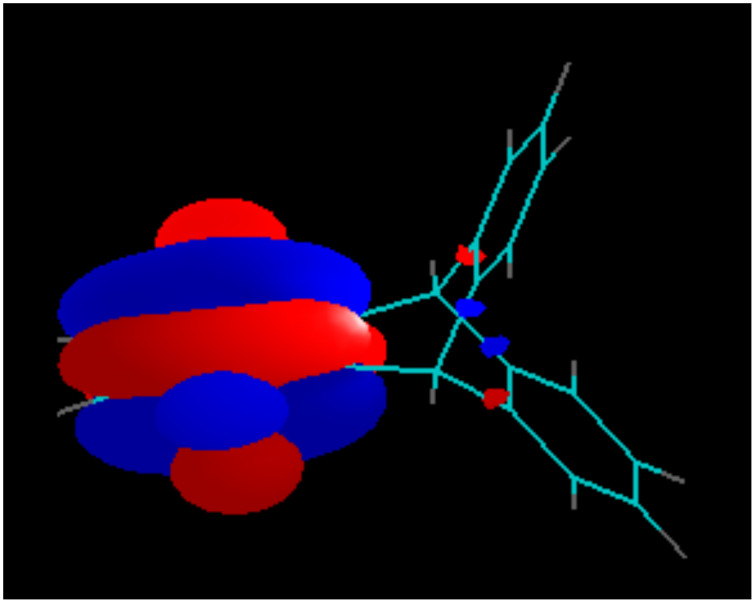
LUMO of Compound **1**.

**Figure 8:**
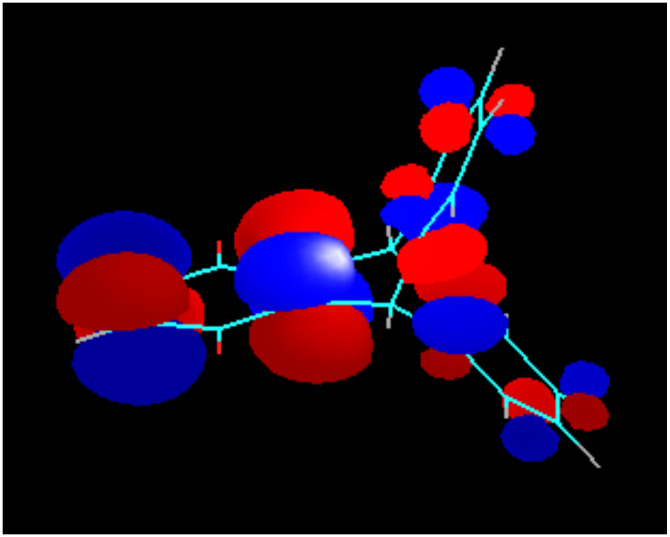
LUMO+1 of Compound **1**.

Due to the Mobius-type transannular orbital overlap in the triptycene-quinone system, each π-orbital of the quinone ring (which is always anti-symmetric with respect to the plane of the ring) may interact only with out-of-phase combinations of the group orbitals of the other two benzene rings. Conversely, interactions of the π-quinone orbitals with the in-phase combinations (they have slightly lower energies, than the out-of-phase combinations and are likely to contribute to the next lower energy molecular orbital) are not allowed by symmetry. This situation is clearly illustrated by the comparison of the calculated LUMO of the triptycene-quinone **1**, lacking noticeable contribution from the non-quinone π-system of the molecule (Figure 7), and the LUMO+1, heavily weighed on the non-quinone benzene rings due to the involvement of the out-of-phase combination of their π-orbitals (Figure 8). Additionally, higher LUMO+1 energy matches better with the antibonding orbital energies of the rest of the bicyclic system.

**Figure 9:**
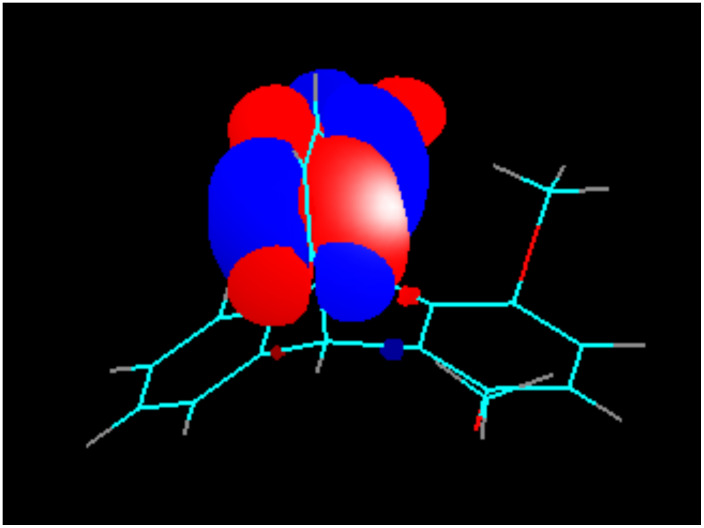
LUMO of Compound **2**.

**Figure 10:**
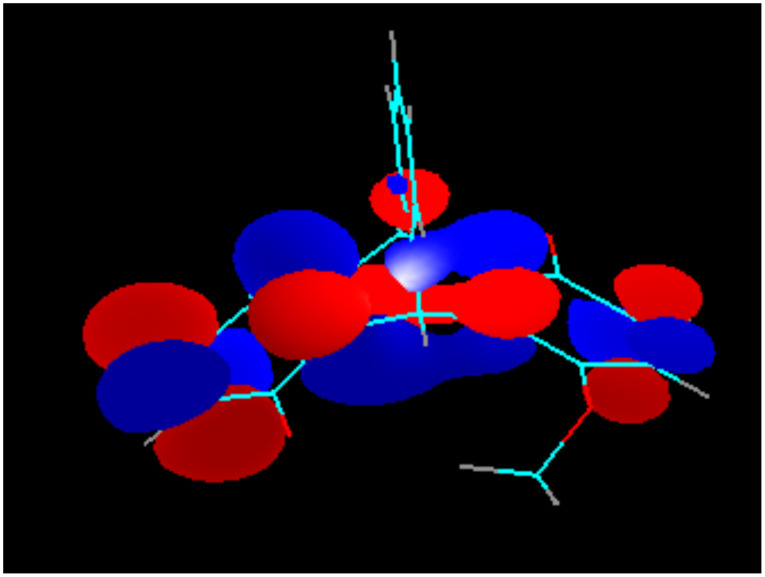
LUMO+1 of Compound **2**.

In the dimethoxy-derivative **2**, the LUMO has some contribution from the bridgehead σ-bonds of the non-quinone part of the molecule, whereas the LUMO+1 orbital is heavily weighted on the substituted benzene ring. This phenomenon is general for the whole series of substituted triptycene-quinones **1–3** and **5** and explains why the substituents in the none-quinone ring influence the second redox-potentials significantly more, than the first redox-potentials. The generality of this orbital overlap pattern is illustrated by Figure 9 and Figure 10.

In the compound **3**, the lone electron pair of the methoxy-group, attached to the quinone ring, makes a major contribution to the LUMO which explains the highest LUMO energy and the lowest redox-potential of **3** in the whole series.

**Figure 11:**
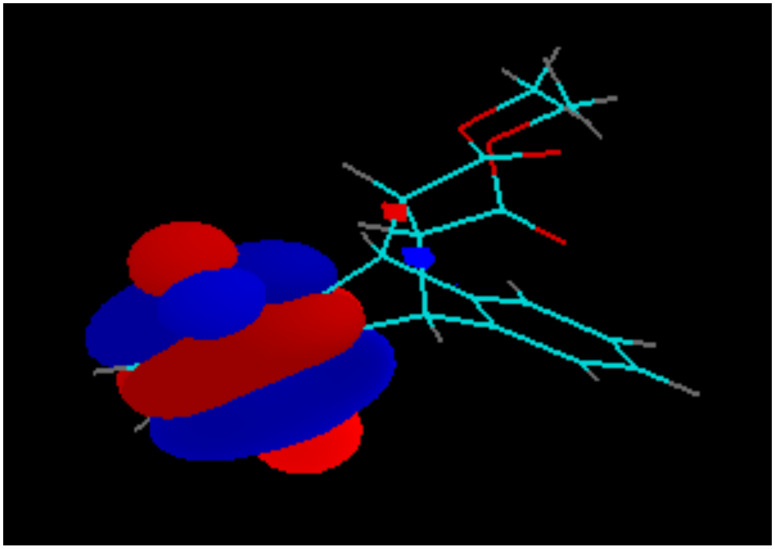
LUMO of Compound **4**.

The non-aromatic fragment with two electron-withdrawing carbomethoxy-groups, attached to the bridgehead σ-bonds and contributing to the LUMO of the quinone **4** (Figure 11), slightly lowers the LUMO energy and hence increases the first redox-potential by 10 mV, compared with the triptycene-quinone **1**.

**Figure 12:**
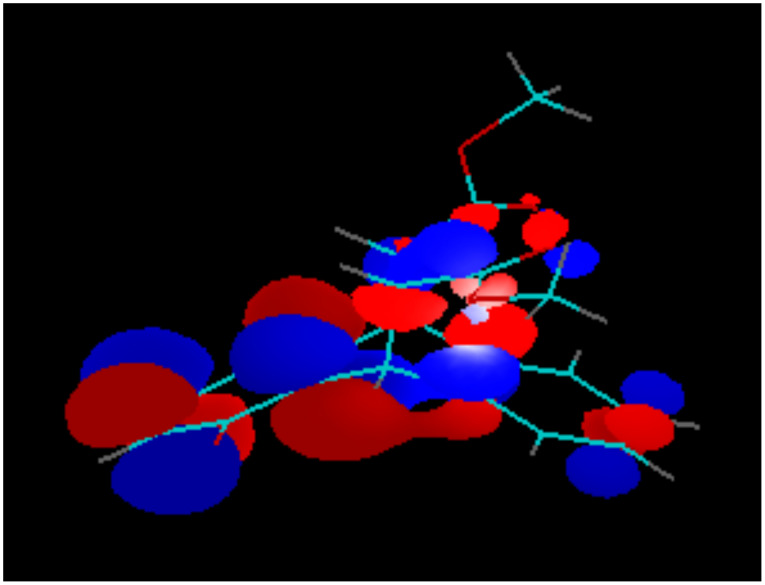
LUMO+1 of Compound **4**.

The significant contribution of the non-aromatic moiety to the LUMO+1 of compound **4** (Figure 12) accounts for the much larger increase (by 50 mV, see Table 1) of the second redox-potential.

**Figure 13:**
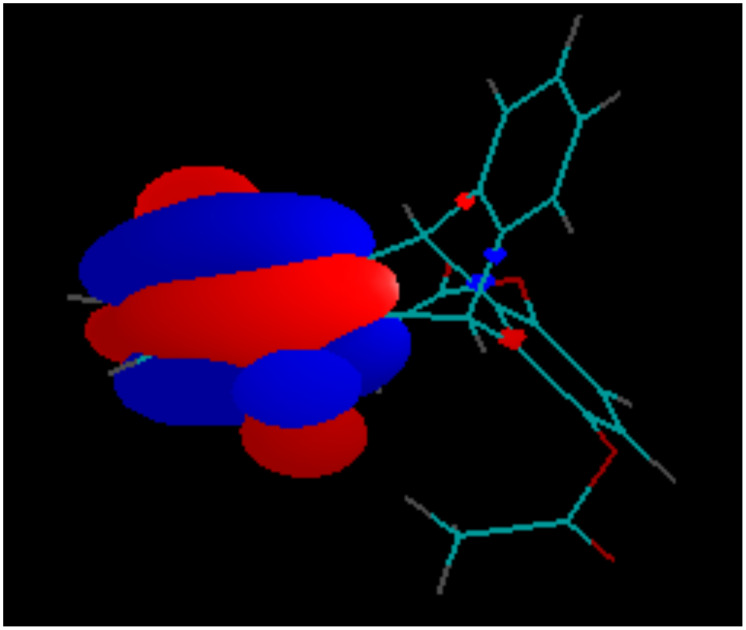
LUMO of Compound **5**.

**Figure 14:**
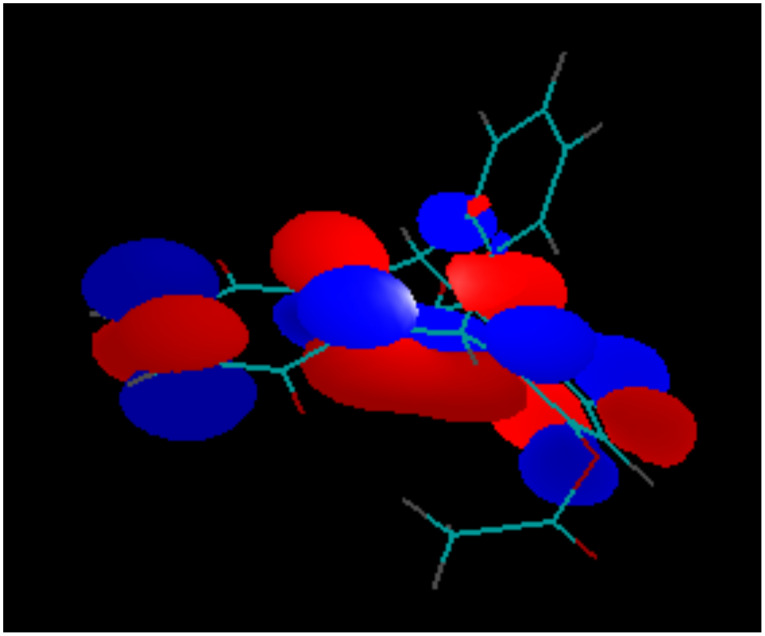
LUMO+1 of Compound **5**.

The different modes of the transannular interaction of orbitals are best illustrated by the different stabilities of the products of one- and two-electron reduction of the 5,8-diacetoxy-derivative **5**. Because of the low contribution of the substituted benzene ring to the LUMO, placing an electron on this orbital does not activate the leaving acetoxy-anions, keeping the product of one-electron reduction stable (Figure 13). The second electron placed on the LUMO+1 orbital of **5**, activates the acetoxy-groups, which causes decomposition of the product of the two electron reduction (Figure 14) and makes the second reduction chemically irreversible.

**Figure 15:**
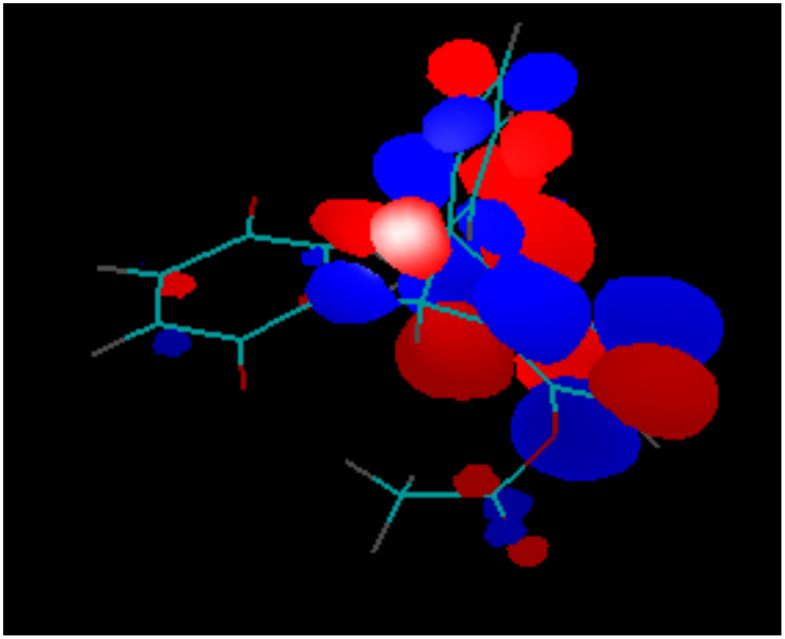
LUMO of the reduced species **5^·−^**.

The LUMO of the reduced species **5^·−^** is also mostly located at the substituted benzene ring (Figure 15), additionally illustrating the reason of the low stability of the dianion **5^2−^**.

## Conclusion

We have shown that first redox-potentials of substituted bicyclic quinones correlate with their calculated LUMO energies and the energies of reduction. The second redox-potentials correlate with calculated LUMO+1 energies. As opposed to the LUMO orbitals, the LUMO+1 orbital coefficients are weighted significantly on the non-quinone part of the bicyclic system. This accounts for: (1) significantly larger substituent effect on the second redox-potentials, than on the first redox-potentials; (2) lack of stability of the product of two electron reduction of 5,8-diacetoxy-9,10-dihydro-9,10-[1,2]benzenoanthracene-1,4-dione **5**.

## Experimental

**Figure 16:**
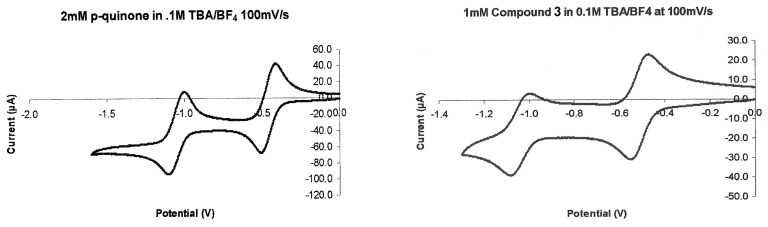
CV for p-benzoquinone and quinone **3**.

9,10-Dihydro-9,10-[1,2]benzenoanthracene-1,4-dione **1** and 2,5,8-trimethoxy-9,10-dihydro-9,10-[1,2]benzenoanthracene-1,4-dione **3** were synthesized as described in the literature [8–9]. 5,8-Dimethoxy-9,10-dihydro-9,10-[1,2]benzenoanthracene-1,4-dione **2** was synthesized as described [2], but with the use of silver oxide in acetone on the last step of oxidation. *cis-anti*-Dimethyl 1,2,3,4-Tetrahydro-1,4-[1,2]benzenonaphthalene-5,8-dione-2,3-dicarboxylate **4** and 5,8-diacetoxy-9,10-dihydro-9,10-[1,2]benzenoanthracene-1,4-dione **5** were synthesized for the first time in our laboratory [10]. A set of redox-potentials was obtained for each of the bicyclic quinones **1–5** by the following procedure. A 2 mmol portion of the compound was dissolved in 25 mL of 0.1 M (n-C_4_H_9_)_4_N^+^BF_4_^−^ (electrochemical grade from Southwestern Analytical) in acetonitrile (HPLC grade) and placed in a three electrode electrochemical cell. The working electrode was a BAS platinum electrode (Bioanalytical Systems, West Lafayette, IN, area ca. 0.02 cm^2^), the auxiliary electrode was a carbon rod and the reference electrode was a BAS Ag/AgCl. To eliminate the influence of oxygen, the solution was degassed with argon gas prior to the experiment and a blanket of argon was maintained over the solution during the experiment. From an initial applied voltage of 0 V, the working electrode's potential was scanned to -1.5 V and then back to 0 V at a rate of 0.1 V/s. For each of the compounds **1–4**, we observed two reduction and two oxidation waves. The formal redox-potentials (E^o'^) were calculated as the average of the complementary peak reduction (Ep_red_) and peak oxidation potentials (Ep_ox_) where (E^o'^ = 1/2(Ep_red_ + Ep_ox_) [11]. In order to check our process, we measured the first reduction potential of *p*-benzoquinone to be -0.507 V, which is exactly the same as the value reported in the literature [3]. The cyclic voltammograms (CV) for p-benzoquinone and the quinone **3** are presented in Figure 16.

For the quinone **5**, lack of a well-defined second oxidation wave indicated that its two electron reduction was chemically irreversible. The potential measurements were not corrected for IR drop. Electrochemical control of the experiment was achieved with a PAR (Princeton Applied Research) model 273 potentiostat equipped with PAR model 270 computer controlled software.

All computational methods were used as implemented to the GAUSSIAN 98W package [12], running on a PC Pentium 4 computer. The molecular structures **1–5** were pre-optimized in vacuum at the AM1 semi-empirical level and then geometry optimized by the B3LYP density functional method (3-21G basis set). The molecular orbitals were calculated at the B3LYP/6-31G** level for the optimized geometries. Consistency of the computational results was checked with a double-split basis set with added diffuse functions (6-311+G**). The product of one-electron reduction of the quinone **5** was treated at the restricted open shell density functional level (ROB3LYP). The MO images were visualized with the Orb Draw 3.00.1 program [13].

The cyclic voltammetry data (first reduction peak potential **E_pr1_**, first oxidation peak potential **E_pox1_**, first formal redox-potential **E^o'^_1_**, second reduction potential **E_pr2_**, second oxidation potential **E_pox2_** and second redox-potential **E^o'^_2_**) for compounds **1–5** are presented in Table 1.

The computed parameters for compounds **1–5** and for their reduced species **1^·−^–5^·−^** are presented in Table 2.

**Table 2:** Computed parameters for compounds **1**–**5** and for their reduced species **1^·−^**–**5^·−^**

**Species**	**E_LUMO_, a.u**.	**E_LUMO+1_, a.u**.	**E_SOMO_, a.u**.	**E, a.u**.	**ΔE_red_, a.u**.

**1**	-0.130	-0.024	-	-919.794	0.065
**1^·−^**	0.099	0.099	0.017	-919.860	
**2**	-0.123	-0.021	-	-1148.839	0.056
**2^·−^**	0.099	0.099	0.030	-1148.894	
2*	-0.137	-0.030	-	-1148.830	0.073
2^·−^*	0.098	0.098	0.007	-1148.902	
**3**	-0.112	-0.010	-	-1263.367	0.052
**3^·−^**	0.100	0.100	0.021	-1263.419	
3*	-0.121	-0.016	-	-1263.359	0.063
3^·−^*	0.100	0.107	0.015	-1263.422	
**4**	-0.132	-0.027	-	-1223.109	0.068
**4^·−^**	0.094	0.094	0.015	-1223.176	
**5**	-0.150	-0.046	-	-1375.532	0.089
**5^·−^**	0.073	0.073	-0.007	-1375.622	

* – Conformation B (Figure 3)
